# Association of physical performance with oral function in older women participating in community‐based health exercise programs

**DOI:** 10.1002/cre2.277

**Published:** 2019-12-28

**Authors:** Sanae Miyoshi, Ayumi Saito, Hideo Shigeishi, Masaru Sugiyama

**Affiliations:** ^1^ Department of Public Oral Health, Program of Oral Health Sciences, Graduate School of Biomedical and Health Sciences Hiroshima University Hiroshima Japan; ^2^ Takehara‐Toyota Dental Hygienists' Association Hiroshima Japan; ^3^ Oral Health Sciences, Graduate School of Biomedical & Health Sciences Hiroshima University Hiroshima Japan

**Keywords:** community‐based health program, older women, oral function, physical function, skeletal muscle mass

## Abstract

**Objectives:**

This study aimed to clarify the relationship between physical performance and oral function in older women participating in community‐based physical and oral exercise programs.

**Material and methods:**

We included 163 older women (mean age: 77.4 ± 8.6 years) who participated in weekly health programs in Takehara City, Hiroshima Prefecture, Japan, from August to December 2018. Physical fitness was assessed using a handgrip strength test, a timed up and go test, a one‐leg standing time with eyes open test, and a 30‐s chair stand test (CS‐30). Tongue pressure, oral diadochokinesis, and dysphagia risk assessment for the community‐dwelling elderly were used to assess oral function.

**Results:**

Participants were divided into women aged 65–74 years (younger group) and those aged ≥75 years (older group). There was no significant difference in oral function measures between the groups. A significant correlation was found between CS‐30 and oral diadochokinesis for /pa/, /ta/, and /ka/ (Spearman's rank correlation; /pa/: *r* = .234, *p* = .009; /ta/: r = .299, *p* = .001; and /ka/: *r* = .283, *p* = .002) in the older group. Multivariable analysis revealed a significant positive relationship between CS‐30 and /ta/ repetition in the older group (*p* = .016).

**Conclusions:**

Oral function (i.e., tongue motor function) may be associated with physical performance (i.e., lower leg muscle strength) in women aged ≥75 years. Further study is necessary to clarify sex differences in oral function deterioration.

## 1 INTRODUCTION

To achieve a healthy life expectancy, it is vital to prevent lifestyle‐related disease and maintain sufficient physical and mental function to continue daily activities. The Japanese Ministry of Health, Labor, and Welfare has implemented a community‐based integrated care system, which can provide community healthcare resources for community‐dwelling older people (Fukutomi, Kimura, Wada, Okumiya, & Matsubayashi, 2013). Thus, comprehensive community‐based health promotion plays an important role in reducing the discrepancy between life expectancy and healthy life expectancy by limiting the need for long‐term care.

Several studies have demonstrated that community‐based educational and health programs improve physical function in community‐dwelling older people in Japan (Kaneko et al., 2009; Miyoshi et al., 2019; Ohara et al., 2015; Sakayori, Maki, Hirata, Okada, & Ishii, 2013). We previously examined a relationship between oral function and long‐term participation in regular physical and oral exercise programs in Japanese community‐dwelling older women and found that older women who had participated in regular physical and oral exercise programs for 3 years or longer did not show a negative relationship between age and oral wetness (Miyoshi et al., 2019). Older women who had participated for less than 3 years showed a reduction in oral wetness with aging (Miyoshi et al., 2019). However, the relationship between physical performance and oral function in participants of health programs has not been fully elucidated. It is speculated that oral function (tongue motor function and swallowing) may be associated with physical function in older people. Maintenance of good oral function may help older people to prevent a decline in body functions and other health problems. Therefore, we performed a cross‐sectional study to investigate the relationship between physical function and oral function in older women who were participating in community‐based health programs in 2018 in Takehara City, Hiroshima.

## 2 METHODS

### 2.1 Participants

We included older people who participated in physical and oral exercise programs every week in a community meeting place in Takehara City, Hiroshima Prefecture, Japan, from August to December 2018. Inclusion criteria were women aged ≥65 years living in Takehara City. Because there were very few male participants in this program, they were not included. Exclusion criteria were people who had been certified by the Japanese long‐term care insurance system as requiring help or long‐term support because we needed to include older people who were competent in the basic activities of daily living (i.e., eating, dressing, toileting, moving, grooming, and bathing). We calculated the sample size required for the correlation coefficient using G*Power (version 3.1.9.4, Heinrich‐Heine‐Universität Düsseldorf, Germany) with a statistical power of 90%, a significance level of 5%, and an effect size of 0.3 to be 112 subjects. Of 189 women aged ≥65 years, seven older women who had been certified as requiring long‐term care and 19 participants who had been certified as requiring help were excluded. Finally, we enrolled 163 women (mean age: 77.4 ± 8.6 years). The study design was approved by the Ethical Committee of Hiroshima University (No. E‐2573), and all participants signed an informed consent agreement. This program mainly consisted of 40 min of physical exercise and 10 min of oral exercise as preventive care (Podsiadlo & Richardson, 1991). The physical exercise (i.e., moderate‐intensity aerobic exercise, strength training, flexibility and balance exercises, and cool‐down activities) was taught by a physical therapist. The oral exercise (i.e., tongue exercise, pursed‐lip breathing, ballooning and pursing exercise of the cheeks, neck rotation exercise, shoulder‐up and ‐down exercise, arm exercise, salivary gland massage, and pronunciation practice) was taught by a dental hygienist. We performed measurement of physical and oral function in all participants after the health program started. On the day of assessment, we investigated the physical and oral performance of participants just before they began the physical and oral exercise.

### 2.2 Measurement of physical performance

We examined physical fitness using a handgrip strength test, a timed up and go test, a one‐leg standing time with eyes open test, and a 30‐s chair stand test (CS‐30). The mean peak handgrip strength of the right and left hands was used for analysis. The timed up and go test is a functional test to assess mobility, falling risk, and walking ability. It requires the participants to stand up from a chair, walk to a line on the floor 3 m away, turn, walk back, and sit down again, and measures the overall time to complete the series of movements (Podsiadlo & Richardson, 1991). The one‐leg standing time with eyes open test is a functional test that requires the participants to raise one leg and stand with their eyes open for a maximum of 60 s. The maximum time for keeping the right or left leg raised was used for evaluation (Japan Ministry of Education,[Link] Culture, Sports, Science, and Technology). The CS‐30 was used to measure the strength of the lower limb muscles. This required the participants to repeatedly stand up from a chair and sit back down as fast as possible for 30 s, and the number of stand‐ups completed was counted (Nakazono, Kamide, & Ando, 2014). Skeletal muscle mass was calculated by bioelectrical impedance analysis using the InBody 230 (InBody Japan, Tokyo, Japan).

### 2.3 Measurement of oral function

We measured maximum tongue pressure using a tongue pressure manometer with a balloon probe (TPM‐01; JMS Co. Ltd., Hiroshima, Japan; Tsuga, Maruyama, Yoshikawa, Yoshida & Akagawa, 2011). The participants were asked to place the balloon probe between the tongue and the anterior part of the palate with the lips closed and to press their tongue against the balloon as hard as possible for 5 s. The mean tongue pressure value was obtained from three independent measurements. Oral diadochokinesis (ODK) was used to evaluate oral motor skills and speech ability. Repetition of the monosyllables /pa/, /ta/, and /ka/ as quickly as possible for 5 s was measured with a device (Kenko‐kun; Takei Scientific Instruments, Niigata, Japan). Repetition of these monosyllables is thought to be associated with lip pressure and tongue motor function (Yamada, Kanazawa, Komagamine, & Minakuchi, 2015). The dysphagia risk assessment for the community‐dwelling elderly (DRACE), which consists of a questionnaire with 12 items, was used to evaluate the risk of dysphagia (Miura, Kariyasu, Yamasaki, & Arai, 2007). The answers consist of “never” (score = 0), “sometimes” (score = 1), and “often” (score = 2). We calculated the total score for each given category and considered a DRACE score ≥ 5 to indicate a risk of dysphagia (Takeuchi et al., 2014).

## 3 STATISTICAL ANALYSIS

Spearman's rank correlation coefficient was used for statistical analysis. The Mann–Whitney *U*‐test was used as a nonparametric alternative to the independent *t*‐test to assess for significant differences, with *p* values of less than .05 regarded to be statistically significant. Fisher's exact test was used to evaluate significant differences in the positive rates of clinical factors between the groups. Multivariable analysis was performed using a forced entry method with each physical function measured as an objective variable. Age, body mass index (BMI), skeletal muscle mass, the number of remaining teeth, and oral function measures with a *p* value of <.05 through univariate analysis were used as explanatory variables. Statistical analysis was performed using JMP® Pro 12 software (SAS Institute Inc., Cary, North Carolina, USA).

## 4 RESULTS

### 4.1 Participants' characteristics

The participants' characteristics are summarized in Table 1. It was speculated that the degree of physical and oral function may be associated with age in older people. Therefore, we divided participants in this study into those aged 65–74 years (younger group) and those aged ≥75 years (older group). BMI and skeletal muscle mass are thought to be an important measure for indicating physical status. Additionally, age‐related tooth loss and denture use may be associated with oral function. Therefore, we examined these clinical factors in both groups. Mean age was 70.4 ± 2.8 in the younger group and 80.3 ± 4.1 in the older group. The mean number of remaining teeth was 22.3 ± 7.4 and 16.1 ± 10.2 in the younger and older groups, respectively. There was a significant difference in mean age, skeletal muscle mass, number of remaining teeth, and denture use between the groups. In measuring physical function, mean handgrip strength was significantly lower in the older group than in the younger group. There was a significant difference in the timed up and go test and the one‐leg standing time with eyes open test between the groups. However, there was no significant difference in tongue pressure, ODK, or DRACE score between the groups.

**Table 1:** Participant characteristics

	Participants aged ≥65 and ≤74 years (*n* = 40)	Participants aged ≥75 years (*n* = 123)	*p* value
Clinical characteristics			
Mean age	70.4 ± 2.8	80.3 ± 4.1	<.001
BMI	22.8 ± 3.2	23.2 ± 3.0	.611
Skeletal muscle mass (kg)	19.8 ± 1.9	18.1 ± 2.2	<.001
Number of remaining teeth	22.3 ± 7.4	16.1 ± 10.2	.001
Denture use			
Non (70)	30	40	<.001^a^
Denture user (93)	10	83	
Physical function measures			
Mean handgrip strength (kg)	23.4 ± 5.0	20.2 ± 3.4	<.001
Timed up and go test (second)	6.3 ± 1.0	7.5 ± 1.8	<.001
One‐leg standing time with eyes open (second)	39.5 ± 21.4	24.8 ± 20.6	<.001
CS‐30 (times)	19.5 ± 4.6	18.4 ± 4.8	.190
Oral function measures			
Tongue pressure(kPa)	32.8 ± 7.4	31.4 ± 7.7	.296
ODK (times/second)			
/pa/	6.3 ± 0.8	6.0 ± 0.8	.091
/ta/	6.1 ± 0.8	5.9 ± 0.9	.181
/ka/	5.9 ± 0.8	5.7 ± 0.8	.174
DRACE score	4.1 ± 3.1	4.1 ± 3.4	.901

*Note*: The Mann–Whitney *U*‐test was used to evaluate significant differences in parameters between the groups.

Abbreviations: CS‐30, 30‐s chair stand test; DRACE, dysphagia risk assessment for the community‐dwelling elderly; ODK, oral diadochokinesis.

a Fisher's exact test was used to evaluate significant differences in clinical factors between the groups.

### 4.2 Correlation between physical performance and oral function in the younger group

The association between physical function measures (i.e., handgrip strength, timed up and go test, CS‐30 score, and one‐leg standing time with eyes open) and oral function measures (i.e., tongue pressure, ODK, and DRACE score) was examined in the younger group (Table 2). Correlation coefficients of *r* = .2–.4 were considered weakly correlated; correlation coefficients of *r* = .4–.7 were considered moderately correlated. There was a significant weak negative correlation between handgrip strength and DRACE score (Spearman's rank correlation; *r* = −.381, *p* = .015). A significant moderate negative correlation between the timed up and go test time and repetition of the monosyllable /ta/ was found (Spearman's rank correlation; *r* = −.403, *p* = .01). Additionally, a significant weak negative correlation was found between the one**‐**leg standing time with eyes open test and the DRACE score (Spearman's rank correlation; *r* = −.335, *p* = .037). A significant positive correlation was found between CS‐30 score and ODK (Spearman's rank correlation; /pa/: *r* = .368, *p* = .019; /ta/: *r* = .409, *p* = .009; and /ka/: *r* = .395, *p* = .012).

**Table 2:** Correlation coefficients between physical performance and oral function in younger participants

	Mean handgrip strength (kg)	Timed up and go test (second)	One‐leg standing time with eyes open (second)	CS‐30 (times)
Tongue pressure (kPa)	0.278	−0.085	0.139	−0.027
ODK (times/second)				
/pa/	0.132	−0.291	0.022	0.368*
/ta/	0.157	−0.403*	0.165	0.409**
/ka/	0.064	−0.156	−0.014	0.395*
DRACE score	−0.381*	0.261	−0.335*	−0.283

*Note*: Spearman's rank correlation coefficient was used for statistical analysis.

Abbreviations: CS‐30, 30‐s chair stand test; DRACE, dysphagia risk assessment for the community‐dwelling elderly; ODK, oral diadochokinesis.

* *p* < .05.

** *p* < .01.

### 4.3 Multivariable analysis for each measure of physical performance in the younger group

Multivariable analysis for each measure of physical function was performed using physical function measures as the dependent variables and oral function measures showing a *p* value of <.05 in Table 2 and clinical factors (i.e., age, BMI, skeletal muscle mass, and the number of remaining teeth) as independent variables in the younger group. Significant independent variables in the multivariable analysis are summarized in Table 3. Handgrip strength was significantly associated with skeletal muscle mass (*p* = .036). There was a significant association between the timed up and go test time and age (*p* = .02). However, no significant association was found between the one‐leg standing time with eyes open test and clinical factors or between the one‐leg standing time with eyes open test and oral function measures. There was also no significant association between CS‐30 and clinical factors or between CS‐30 and oral function measures.

**Table 3:** Multivariable analysis for each measure of physical performance in younger participants

	Variable	B	SE	*t*	*p* value	95% CI
Mean handgrip strength	Skeletal muscle mass	1.039	0.465	2.234	.036	(0.074, 2.003)
Timed up and go test	Age	0.205	0.082	2.499	.020	(0.035, 0.376)

*Note*: Multivariable analysis was performed using a forced entry method. *p* values of less than .05 were regarded as statistically significant.

### 4.4 Correlation between physical performance and oral function in the older group

There were significant weak positive correlations between handgrip strength and tongue pressure (Spearman's rank correlation; *r* = .277, *p* = .002) and between handgrip strength and ODK (/pa/: *r* = .202, *p* = .025; /ta/: *r* = .204, *p* = .024; and /ka/: *r* = .229, *p* = .011; Table 4). A significant weak negative correlation between the timed up and go test time and ODK was found (Spearman's rank correlation; /pa/: *r* = −.270, *p* = .003; /ta/: *r* = −.295, *p* = .001; and /k/a: *r* = −.337, *p* < .001). Additionally, there was a significant weak positive correlation between the one**‐**leg standing time with eyes open test and ODK (Spearman's rank correlation; /pa/: *r* = .214, *p* = .018; /ta/: *r* = .281, *p* = .002; and /ka/: *r* = .250, *p* = .006). Furthermore, a significant weak correlation was found between CS‐30 score and ODK /pa/, /ta/, and /ka/ (Spearman's rank correlation; /pa/: *r* = .234, *p* = .009; /ta/: *r* = .299, *p* = .001; and /ka/: *r* = .283, *p* = .002).

**Table 4:** Correlation coefficients between physical performance and oral function in older participants

	Mean handgrip strength (kg)	Timed up and go test (second)	One‐leg standing time with eyes open (second)	CS‐30 (times)
Tongue pressure (kPa)	0.277**	−0.165	−0.024	0.069
ODK (times/second)				
/pa/	0.202*	−0.270**	0.214*	0.234**
/ta/	0.204*	−0.295**	0.281**	0.299**
/ka/	0.229*	−0.337***	0.250**	0.283**
DRACE score	−0.017	0.092	−0.115	−0162

*Note*: Spearman's rank correlation coefficient was used for statistical analysis.

Abbreviations: CS‐30, 30‐s chair stand test; DRACE, dysphagia risk assessment for the community‐dwelling elderly; ODK, oral diadochokinesis.

* *p* < .05.

** *p* < .01.

*** *p* < .001.

### 4.5 Multivariable analysis for each measure of physical performance in the older group

Significant independent variables in the multivariable analysis are summarized in Table 5. Handgrip strength was significantly associated with skeletal muscle mass (*p* = .003; Table 5). There was a significant association between the timed up and go test time and age (*p* < .001). A significant negative association was found between one‐leg standing time with eyes open and age (*p* < .001). There was a significant relationship between CS‐30 score and repetition of /ta/ (*p* = .016).

**Table 5:** Multivariable analysis for each measure of physical performance in older participants

	Variable	B	SE	*t*	*p* value	95% CI
Mean handgrip strength	Skeletal muscle mass	0.536	0.176	3.051	.003	(0.186, 0.885)
Timed up and go test	Age	0.157	0.042	3.690	<.001	(0.072, 0.241)
One‐leg standing time with eyes open	Age	−2.084	0.526	−3.965	<.001	(−3.130, −1.037)
CS‐30	/ta/	2.778	1.124	2.472	.016	(0.540, 5.016)

*Note*: Multivariable analysis was performed using a forced entry method. *p* values of less than .05 were regarded as statistically significant.

Abbreviation: CS‐30, 30‐s chair stand test.

## 5 DISCUSSION

In this study, older participants exhibited significantly lower skeletal muscle mass than younger participants. Muscle strength (i.e., handgrip strength) was also lower in older participants than in younger participants. Our results suggest that the decline in skeletal muscle volume (i.e., sarcopenia) and decreased muscle strength may be associated with aging.

It was speculated that older participants would show greater deterioration in oral function than younger participants. However, there was only a small difference in the decline in tongue pressure, ODK, and DRACE score in the older group when compared with the younger group. This result suggests that not age but other clinical factors may be involved in the maintenance of oral function in older women. Wakasugi, Tohara, Machida, Nakane, and Minakuchi (2017) reported that men aged ≥65 years showed apparent deterioration of oral function with aging. In contrast, older women exhibited a slow decrease in tongue pressure with aging, and there was no significant correlation between tongue pressure and physical functions such as handgrip strength and walking speed (Wakasugi et al., 2017). These observations indicate that oral function can be maintained in older women even when their physical function is impaired to some degree with aging. Although the reasons for the differences in oral function deterioration between men and women remain unknown, increased loss of skeletal muscle (e.g., oropharyngeal muscle) in men may play a part (Logemann et al., 2000; Logemann, Pauloski, Rademaker, & Kahrilas, 2002). Importantly, such sex differences should be considered when designing oral health programs and when assessing the deterioration of oral function in community‐dwelling older people.

When assessing clinical factors related to physical function, we found a significant association between handgrip strength and skeletal muscle mass by multivariable analysis in both groups. The findings of this study are consistent with previous research showing a close relationship between skeletal muscle mass and handgrip strength in middle‐aged and older women (de Souza et al., 2017). Additionally, the timed up and go test time increased significantly with aging in both the younger and older groups, suggesting that impairment of mobility and walking capacity is associated with age. In evaluating the relationship between physical performance and oral function, we observed that the CS‐30 score was significantly associated with repetition of the monosyllable /ta/ in the older group. The CS‐30 is a useful method for evaluating lower limb muscle strength in a clinical setting (Góes et al., 2012). Repetition of the syllable /ta/ is reported to be related to lingual masticatory function and tongue pressure (Kikutani et al., 2009; Yamada et al., 2015). It is likely that lower limb muscle strength is associated with tongue motor function in women aged ≥75 years. It has been reported that lower limb muscle mass is significantly associated with poor physical function (i.e., walking and repeated chair stands) in older people (Visser, Deeg, Lips, Harris, & Bouter, 2000). Additionally, the one‐leg standing time with eyes open test, which is used to assess lower limb muscle strength, was significantly associated with repetition of the monosyllable /ka/ in older people (Izuno et al., 2016) and was also positively associated with bite force and the number of remaining teeth (Iinuma et al., 2012; Yoshida et al., 2009). These results support the theory that there is a strong relationship between tongue function and lower leg muscle strength in older women. Impairment of tongue function may be a predictive risk factor for falls in older women due to decreased lower limb strength.

The significant risk factors for sarcopenia‐associated dysphagia are considered to include a decline in skeletal muscle (i.e., swallowing‐related muscles), low activity in daily living, and poor nutrition (Fujishima et al., 2019). In this study, the mean DRACE score was less than 5 in both the younger and older groups, indicating that many participants were at a low risk of dysphagia. Our exercise program including swallowing muscle training may have been related to the improvement and maintenance of the participants' swallowing function. The nutritional status of the participants was not assessed in our community‐based health programs. Individual assessment of nutritional status would be required to provide effective nutritional guidance for participants suffering from malnutrition. Additionally, systemic disease is thought to be a significant factor associated with physical function. However, this study did not include the collection of detailed information from participants about systemic disease and medication. Therefore, further investigation is likely to be necessary to clarify any correlation between physical function and systemic disease or medication.

One limitation of the present study should be noted. We focused on older individuals who participated in health programs; we did not examine physical and oral function in people not participating in such programs. Accordingly, additional study of older individuals who do not participate in such programs is required to generalize our obtained results (i.e., significant relationship between physical and oral function).

Our previous study revealed that regular physical and oral exercise may be helpful for older women who have not been certified as requiring long‐term care to prevent the impairment of oral function (Miyoshi et al., 2019). Given that social frailty (i.e., living alone, going outside less frequently, and having few opportunities to talk with others) is associated with the onset of disability in older adults (Makizako et al., 2015), participation in health programs may be related to the maintenance of psychological health by promoting social activity. Therefore, it is essential to continue to provide community healthcare resources for community‐dwelling older people.

## 6 CONCLUSION

Oral function (i.e., tongue motor function) was importantly associated with physical performance (i.e., lower leg muscle strength) in women aged ≥75 years. Multivariate analysis of the small number of participants in the younger group may have resulted in a possible bias. Therefore, further additional study is necessary to clarify the correlation between physical and oral function in people aged 65–74 years. Moreover, independent older women may exhibit a slow decline in oral motor function. Further additional study will be necessary to clarify the sex differences in the deterioration of oral function in community‐dwelling older adults.

## CONFLICT OF INTEREST

The authors declare that they have no conflict of interest.
